# Obesity impacts placental function through activation of p-IRE1a-XBP1s signaling

**DOI:** 10.3389/fcell.2023.1023327

**Published:** 2023-02-01

**Authors:** Wei-Bin Shen, Bingbing Wang, Ruofan Yao, Katherine R. Goetzinger, Sheng Wu, Haijun Gao, Peixin Yang

**Affiliations:** ^1^ Department of Obstetrics, Gynecology and Reproductive Sciences, University of Maryland School of Medicine, Baltimore, MD, United States; ^2^ Center for Metabolic Disease Research, Department of Cardiovascular Sciences, Temple University School of Medicine, Philadelphia, PA, United States; ^3^ Departmentof Physiology and Biophysics, Howard University College of Medicine, Washington, DC, United States

**Keywords:** placenta, maternal obese, endoplasmic reticulum stress, IRE1α, XBP1S, chaperones

## Abstract

Maternal obesity is associated with a variety of obstetrical outcomes including stillbirth, preeclampsia, and gestational diabetes, and increases the risk of fetuses for congenital heart defects. Obesity during pregnancy represents a major contribution to metabolic dysregulation, which not only plays a key role in the pathogenesis of adverse outcome but also can potently induce endoplasmic reticulum (ER) stress. However, the mechanism associating such an obesogenic metabolic environment and adverse pregnancy outcomes has remained poorly understood. In this study, we aimed to determine whether the ER stress pathways (also named unfolded protein response (UPR)) were activated in the placenta by obesity. We collected placenta from the obese pregnancy (n = 12) and non-obese pregnancy (n = 12) following delivery by Caesarean-section at term. The specimens were assessed with immunocytochemistry staining and RT-QPCR. Our results revealed that in the obese placenta, p-IRE1α and XBP1s were significantly increased, CHOP and nine UPR chaperone genes were upregulated, including GRP95, PDIA6, Calnexin, p58IPK, SIL-1, EDEM, Herp, GRP58 and Calreticulin. However, Perk and BiP are not activated in the obese placenta. Our data suggest that upregulated p-IRE1α and XBP1s signaling, and UPR chaperone genes may play an important role in maternal obesity-induced placental pathology. In conclusion, this is the first report on ER stress and UPR activation in the placenta of maternal obesity. Our findings represent the first step in the understanding of one of the key ER signaling pathways, also referred to IRE1α-XBP1, in placental pathophysiology affected by obesity, which may be an important mechanism accounting for the observed higher maternal and perinatal risks.

## Introduction

Modern society is continuing to face the increasing challenge of the elevated incidence rate of obesity, which presents a severe detriment to human health as well as a major cause of morbidity and mortality across generations (Brombach et al., 2022). Maternal obesity is significantly associated with a variety of obstetrical outcomes including stillbirth, preeclampsia, and gestational diabetes (Rubens et al., 2022). Furthermore, fetuses are at increased risk for congenital birth defects and offspring are more likely to develop cardiometabolic diseases in later life (Whitaker et al., 1997; Blomberg and Kallen, 2010; Reynolds et al., 2013; Gaillard et al., 2014a; Gaillard et al., 2014b).

Maternal obesity is typically characterized with alterations in circulating levels of various substances including hormones, nutrients, growth factors, cytokines, and lipids. However, the mechanisms associated with such an obesogenic metabolic environment and short- and long-term adverse pregnancy outcomes have remained poorly understood (Kelly et al., 2020). The placenta, constituting the interface between the mother and fetus, plays a key role in mediating nutrient and gas exchange to sustain and ensure a successful pregnancy (Jansson and Powell, 2013). Mounting evidence supports that maternal obesity is a leading cause of extensive changes in placental function, which predominantly accounts for maternal obesity-derived adverse effects on fetal development (Kelly et al., 2020). While the research on fetal and maternal outcomes has been well conducted, however, investigation on the effects of maternal obesity effects on the placenta and their subsequent link with obstetrical outcomes has remained relatively limited.

Obesity during pregnancy represents a major contributor to metabolic dysregulation, which not only plays a key role in the pathogenesis of adverse outcomes but also can potently induce endoplasmic reticulum (ER) stress (Park et al., 2020). ER is the major site allowing the folding and maturation of proteins. ER stress can be detected in nearly all tissues with elevated metabolic and endocrine demands in obese individuals. Nevertheless, under an obesogenic condition, ER stress is largely dependent on insulin resistance in adipose tissues. ER stress-response pathways, also known as the unfolded protein response (UPR), are comprised of three highly conserved signaling pathways: ATF6 and IRE1-XBP1 pathways that enhance folding capacity by upregulating the activity of ER chaperones such as GRP78 and heat shock protein 90 kDa beta ember 1 (GRP94) and promote phospholipid synthesis, and PERK-eIF2α pathway that attenuates non-essential protein synthesis (Yung et al., 2014; Adams et al., 2019). Furthermore, the ER-associated protein degradation (ERAD) pathway functions by promoting protein degradation (Schroder and Kaufman, 2005).

A number of studies have explicitly demonstrated that cell signaling downstream inflammatory, metabolic, and oxidative stress mediates the effects of maternal diabetes on placental function (Brombach et al., 2022). In this study, we focused on ER stress and UPR activation in the placenta from a pregnancy complicated with maternal obesity. We used a variety of biochemical approaches including immunohistochemistry and quantitative PCR to accomplish our goal.

## Materials and methods

### Study design

As described in our previous study (Yao et al., 2022), patients admitted for scheduled cesarean delivery at the University of Maryland Medical Center were enrolled for this study. The enrollment criteria: patients at least 18 years old, at least 37 weeks gestational age based on clinical dating criteria; unlabored and scheduled for cesarean delivery; and patients without a diagnosis of diabetes or hypertension, chorioamnionitis, abnormal liver function or fetal growth restriction. Patients were classified as non-obese if pre-pregnancy maternal BMI was between 18.5 and 24.9 kg/m^2^ or obese if pre-pregnancy BMI was greater than or equal to 30.0 kg/m^2^. A total of 24 subjects (12 normal-weight and 12 obese patients) were enrolled from October 2016 through December 2017. This study was approved by the University of Maryland Internal Review Board (IRB# HP-00066908).

### Reverse transcription-quantitative PCR (RT-qPCR)

Total RNA was isolated from the placenta using a RNeasy minikit (Qiagene, Valencia, CA), reverse transcribed using the QuantiTect Reverse Transcription kit (Qiagen, Valencia, CA), and followed by QPCR. QPCR were performed using PowerUP Sybr Green Master Mix (Thermofisher Scientific, Waltham, MA; Cat #A25779). QPCR primer sequences for XBP1s, CHOP, and UPR chaperone genes were listed in Table 1.

**TABLE 1:** Primers used for the QPCR.

Gene		Primer sequences (5’-->3′)	Accession#	Sources: PrimerBank
CHOP	Forward	GCA​CCT​CCC​AGA​GCC​CTC​ACT​CTC​C	NM_001195053	Yang et al. (2016)
	Reverse	GTC​TAC​TCC​AAG​CCT​TCC​CCC​TGC​G		
Bip	Forward	CGA​GGA​GGA​GGA​CAA​GAA​GG	X87949	Yang et al. (2016)
	Reverse	CAC​CTT​GAA​CGG​CAA​GAA​CT		
Calnexin	Forward	CCT​TCC​TGT​GTT​CCT​GGT​TAT​C	M94859	Dong et al. (2018)
	Reverse	TCC​TTC​TCT​TCT​TCC​TCT​TCC​T		
GRP94	Forward	GCT​GAC​GAT​GAA​GTT​GAT​GTG​G	NM_003299	Dong et al. (2018)
	Reverse	CAT​CCG​TCC​TTG​ATC​CTT​CTC​TA		
PDIA6	Forward	GGA​CAC​TGC​AAA​AAC​CTA​GAG​C	NM_001282704	Dong et al. (2018)
	Reverse	CCA​GAA​CCT​GAT​TGA​CTG​TAG​CA		
XBP1s	Forward	GCTGAGTCCGCAGCAGGT	NM_006931	221136810c1
	Reverse	CTG​GGT​CCA​AGT​TGT​CCA​GAA​T		
XBP1	Forward	GGT​CTG​CTG​AGT​CCG​CAG​CA	NM_001042	83722278c1
	Reverse	AAG​GGA​GGC​TGG​TAA​GGA​AC		
Calreticulin	Forward	CCT​GCC​GTC​TAC​TTC​AAG​GAG	NM_004343	209862753c1
	Reverse	GAA​CTT​GCC​GGA​ACT​GAG​AAC		
GRP58	Forward	GCC​TCC​GAC​GTG​CTA​GAA​C	NM_005313	67083697c1
	Reverse	GCG​AAG​AAC​TCG​ACG​AGC​AT		
SIL-1	Forward	AGG​CAA​AAC​TCC​AAT​ATG​AGG​AC	NM_022464	83641898c2
	Reverse	GAT​GTG​TAG​GTG​TTG​GTG​TTG​AT		
P58IPK	Forward	GGA​TGC​AGA​ACT​ACG​GGA​ACT	NM_006260	300192905c3
	Reverse	TCT​TCA​ACT​TTG​ACG​CAG​CTT		
Herp	Forward	TGC​TGG​TTC​TAA​TCG​GGG​ACA	NM_001010990	58530858c2
	Reverse	CCA​GGG​GAA​GAA​AGG​TTC​CG		
EDEM	Forward	CGG​ACG​AGT​ACG​AGA​AGC​G	NM_014674	197304767c1
	Reverse	CGT​AGC​CAA​AGA​CGA​ACA​TGC		
ERp72	Forward	GGC​AGG​CTG​TAG​ACT​ACG​AG	NM_004911	157427676c1
	Reverse	TTG​GTC​AAC​ACA​AGC​GTG​ACT		
GAPDH	Forward	ACA​ACT​TTG​GTA​TCG​TGG​AAG​G	NM_001256799	378404907c2
	Reverse	GCC​ATC​ACG​CCA​CAG​TTT​C		

### Immunocytochemistry staining

Formalin-fixed, paraffin-embedded (FFPE) placenta tissues were sectioned. The immunocytochemistry staining was performed as described (Xu et al., 2021; Shen et al., 2022). The sections were deparaffined and rehydrated followed by antigen retrieval (Citrate buffer, pH6) and H_2_O_2_ treatment (2%, 15 min). Sections were incubated in blocking solution (phosphate-buffered saline (PBS) containing 4% normal goat serum, 0.2% Triton X-100) for 1 h, then with the primary antibodies diluted in block solution at 4°C overnight. Sections were incubated with biotinylated secondary antibody for 1 h and followed by 1 h with ABC solution (VectorLab, Elite Kit, Cat# PK6100, 1:500). Antibody labeling was visualized in diaminobenzidine (DAB, 10 mg/50 mL) chromogen in 0.175 M sodium acetate +0.003% H_2_O_2_ (brown products). Sections were counterstained with hematoxylin, dehydrated and coverslipped. Images were captured using a Nikon Eclipse Ni microscope (Nikon Instrument Inc., NY, United States).

Following antibodies were used in this study: rabbit anti-p-IRE1α antibody (Thermofisher Scientific, Cat #PA1-16927, 1:2,000); rabbit anti- XBP1s antibody (ProteinTech, Cat #24868-1-AP, 1:2,000); Rabbit anti-p-Perk antibody (Thermofisher Scientific, Cat #PA5-37773, 1:2,000); and rabbit anti-Bip antibody (Cell Signaling Technology, Cat #CST-31776, 1:2,000).

The immunocytochemistry staining was quantified with the use of ImageJ program, an open-source software for processing and analyzing images (Schneider et al., 2012). The percentage of staining area by DAB immunocytochemistry was derived with normalization to the total area with nuclear hematoxylin staining according to the instructions as specified by this program, and the mean was obtained from three or four random fields in the same cross-section of each sample. A total of ten placenta samples were from non-obese pregnancies (controls) and 12 samples were from obese pregnancies.

### Statistical analysis

Maternal obesity was recorded as described (Yao et al., 2022). Briefly, maternal pre-pregnancy weight was obtained from medical records of the last recorded weight within 1 year prior to pregnancy. If this was not available, the earliest weight recorded within the first trimester was used. Maternal height was obtained from prenatal records. Pre-pregnancy BMI was calculated as the weight (kg) divided by the square of height m). Based on BMI information, the patients were categorized as non-obese (normal-weight, BMI: 18.5–24.9 kg/m^2^) or obese (BMI ≥30.0 kg/m^2^).

Images from the immunostaining and XBP1s/XBP1 agarose gel were quantified with ImageJ. The data from non-obese were compared with the obese group using non-parametric Mann-Whitney *U*-test. All statistical analyses were performed using Graphpad prism, Ver 5.0 (GraphPad Software, San Diego, CA).

## Results

### Maternal obesity activates p-IRE1a-XBP1s but not p-Perk signaling in the placenta

**FIGURE 1:**
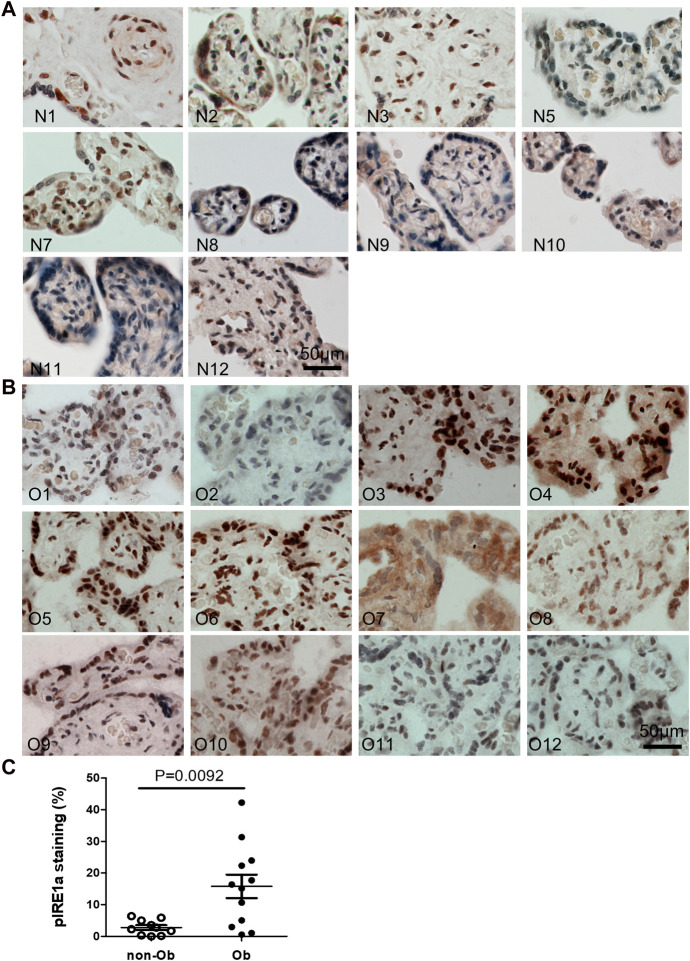
Maternal obesity induces IRE1α phosphorylation in the placenta. Placenta FFPE sections were labeled with anti-p-IRE1α specific antibody in the non-obese group **(A)**, n = 10) and the obese group **(B)**, n = 12). The labeling intensity was semi-quantified with ImageJ **(C)**, showing the increased p-IRE1α in the obese placenta. Non-parametric Mann-Whitney *U*-test with *p* < 0.05 indicates a significant difference in p-IRE1α between the non-obese group to the obese group. Values are mean ± SEM. N1-N12: non-obese samples; O1-O12: obese samples.

**FIGURE 2:**
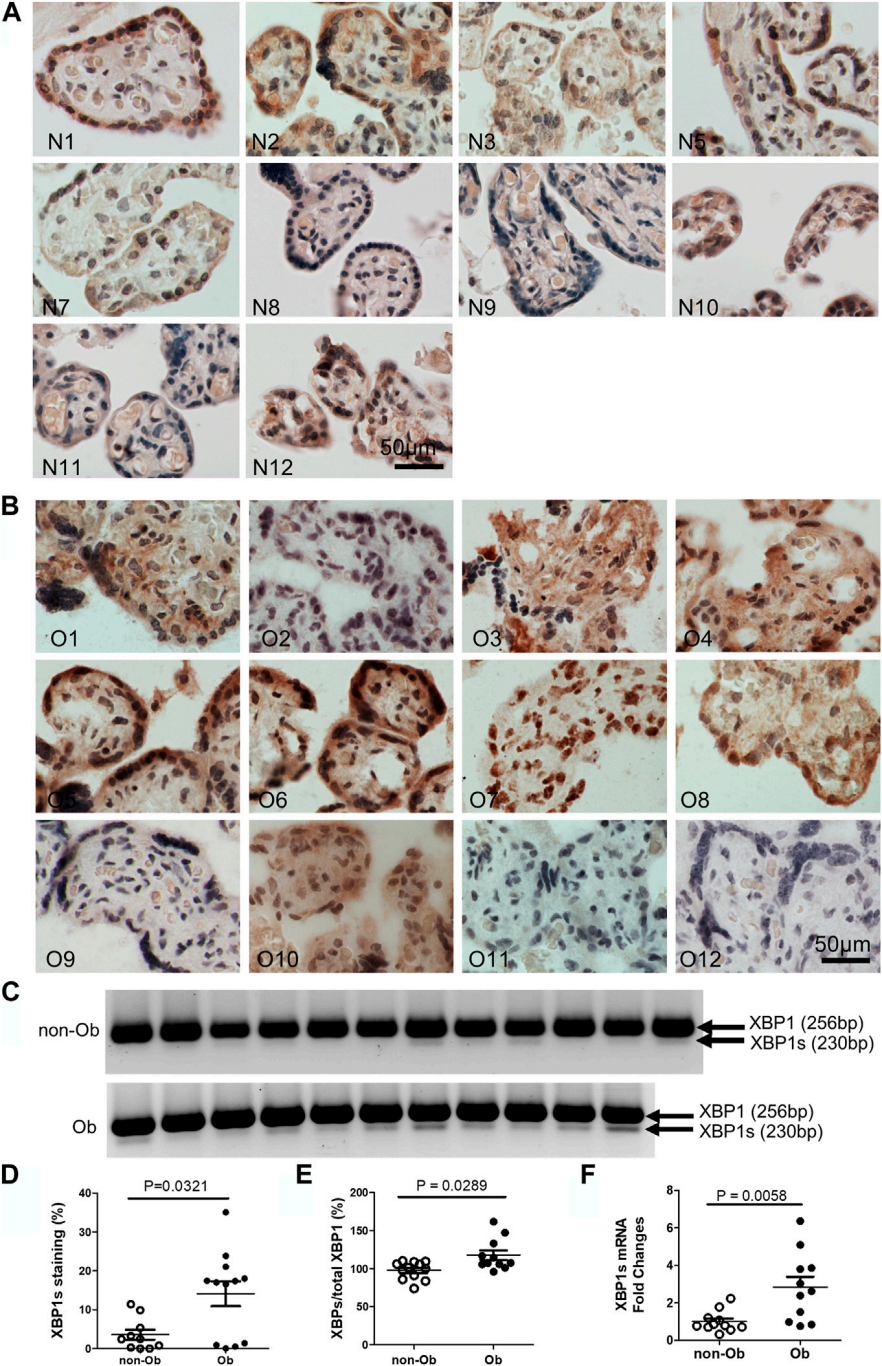
Obesity activates XBP1 splicing in the placenta. XBP1s specific antibody was used to label activated XBP1s. Compared to the non-obese placenta **(A)**, n = 10), the obese placenta exhibited increased XBP1s **(B)**, n = 12). Elevated XBP1s was visualized by PCR with a primer pair flanking splicing regions followed by electrophoresis **(C)** (unspliced, 256 bp; spliced XBP1, 230 bp) (Yoon et al., 2019). Quantified with ImageJ on the immunocytochemically labeled sections **(D)** or XBP1s PCR gel images **(E)** showed increased XBP1s in the obese placenta. Furthermore, Reverse Transcription (RT)-QPCR demonstrated increase of XBP1s in the obese placenta **(F)**. Non-parametric Mann-Whitney *U*-test was used to compare the XBP1s between the non-obese and obese placenta. Values are mean ± SEM. N1-N12: non-obese samples; O1-O12: obese samples.

**FIGURE 3:**
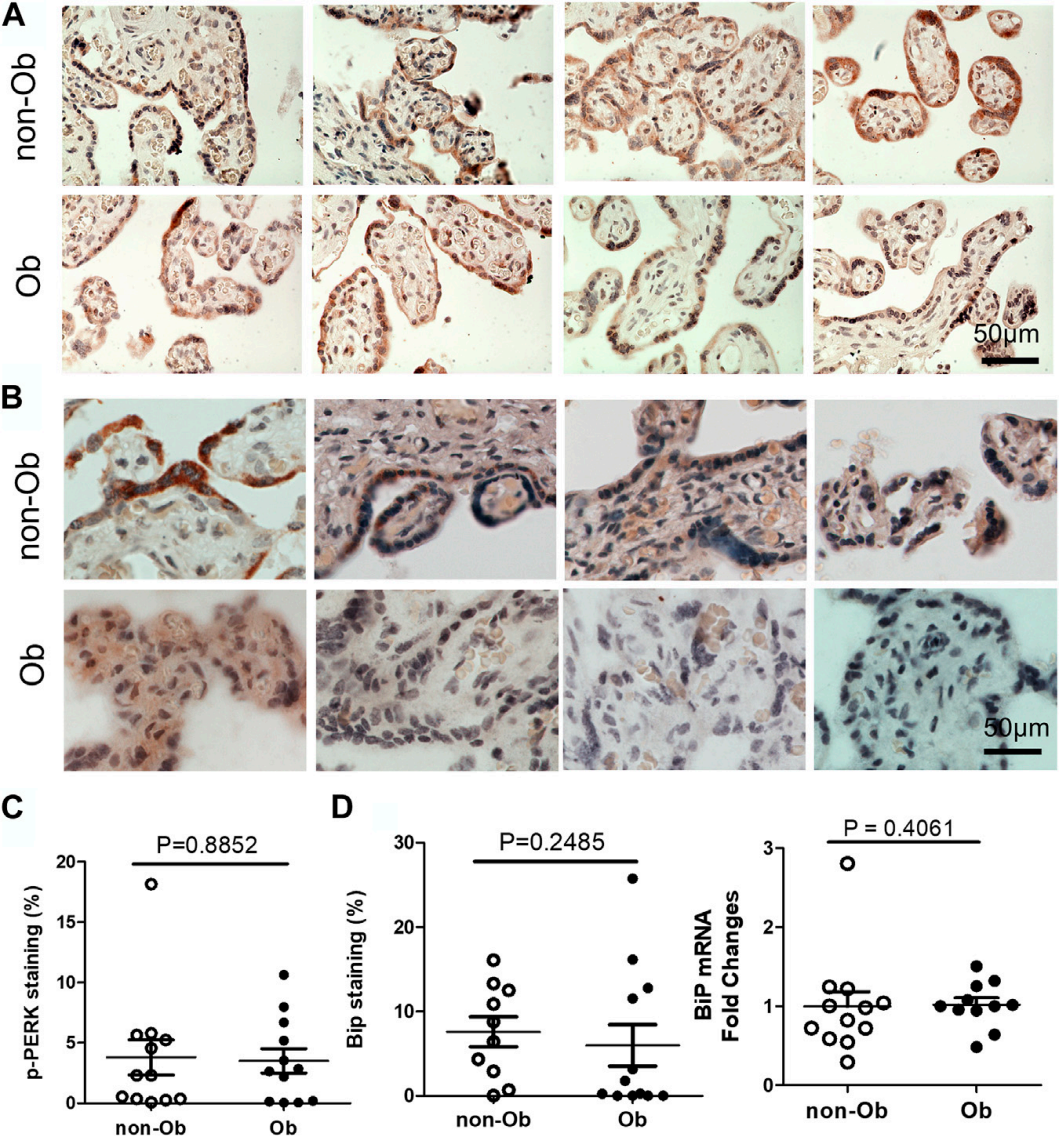
Obesity does not activate Perk signaling and BiP in the placenta. p-Perk was at similar levels in the non-obese (n = 12) and obese placenta (n = 12). Four representative p-Perk labeled images from either non-obese or obese were showed **(A)**. One of the chaperone gene, BiP, were at similar intensity in the non-obese and obese placenta (non-obese, n = 10; obese, n = 12). **(B)**, Four representative BiP labeled images were shown from each group. Quantification of p-Perk **(C)** or BiP **(D)** labeling was performed with ImageJ. Non-parametric Mann-Whitney *U*-test was used to compare the controls to others. Values are mean ± SEM.

Excessive ER stress disrupts homeostasis in the placenta and contributes to the pathology of human pregnancy complications (Mizuuchi et al., 2016; Nakashima et al., 2019). Maternal obesity impacts both the placenta and the fetus (Howell and Powell, 2017). To Investigate the role of ER stress in maternal obesity-induced placental dysfunction, we assessed two ER stress markers—p-IRE1α and p-Perk on the placental FFPE sections by Immunocytochemistry. As shown in Figure 1, p-IRE1α was significantly increased in both syncytiotrophoblast and cytotrophoblasts in the obese placenta compared to non-obese controls. Upon activation, IRE1α is phosphorylated and cleaves its downstream target XBP1 to form spliced XBP1s mRNA. The translated XBP1s proteins translocated into the nucleus in which XBP1s activates transcription of ER chaperone genes (Yoshida et al., 2006). Indeed, placenta from the maternal obesity subjects showed increased XBP1s levels in both the cytosol and nucleus (Figures 2A–D). Electrophoresis of XBP1/XBP1s PCR products demonstrated increased XBP1s in the obese placenta (Figures 2C–E). Furthermore, RT-QPCR with XBP1s specific primer pair demonstrated 2.8-fold increase of XBP1s (Figure 2F). p-Perk protein was visualized with immunostaining in the cytosol of the syncytiotrophoblast. However, there is no difference of p-Perk intensity between the non-obese and the obese placenta (Figures 3A–C), suggesting Perk signaling may not impacted in the obese pregnancy.

### Maternal obesity upregulates CHOP and ER chaperones in the placenta

**FIGURE 4:**
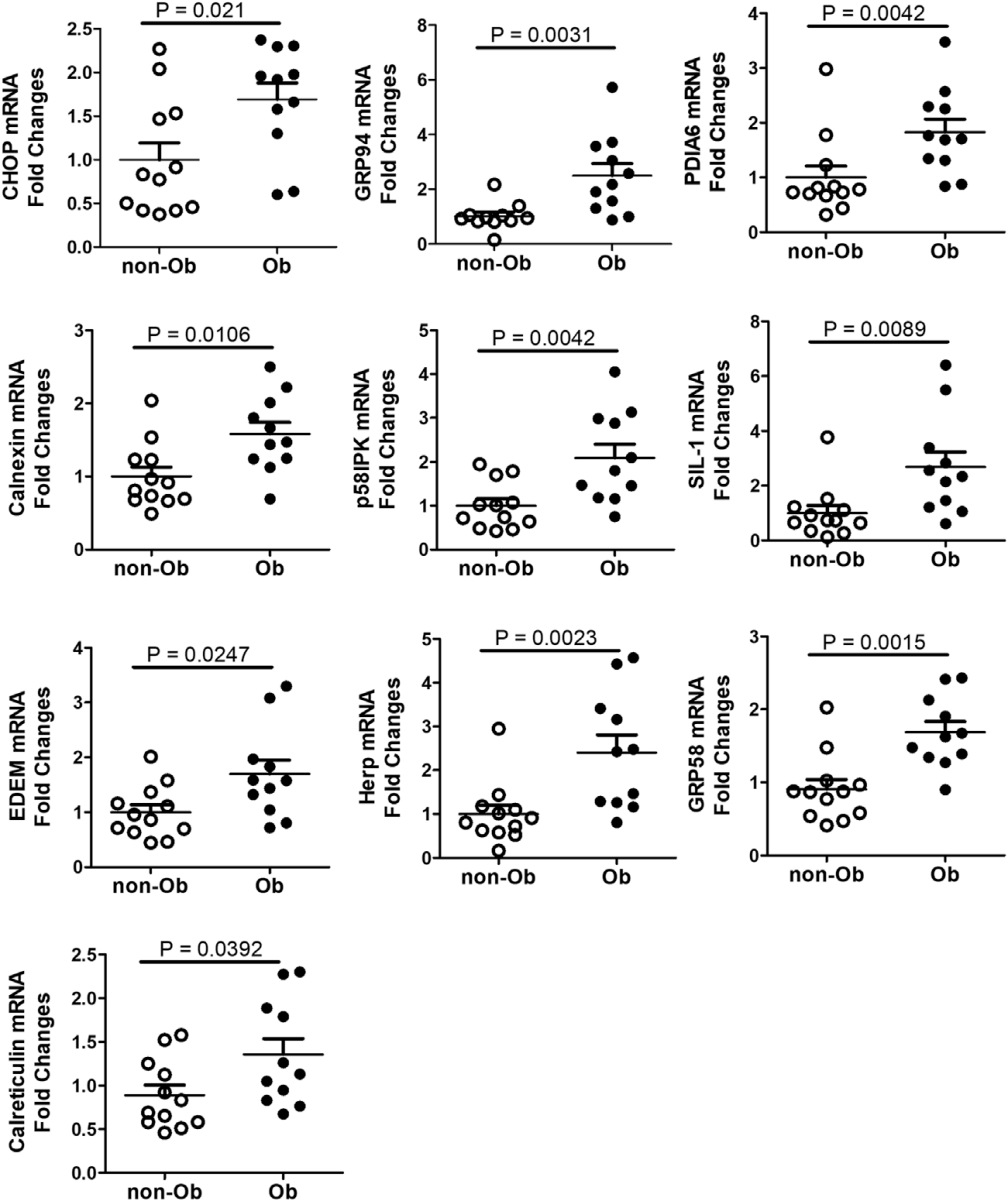
CHOP and UPR chaperones were upregulated in the placenta of obese pregnancy. CHOP was upregulated in obese placenta. Nine out of ten UPR chaperone genes we assessed with RT-QPCR were upregulated in the obese placenta. Non-parametric Mann-Whitney *U*-test was used to compare the chaperone gene expression in the non-obese placenta with the obese placenta, and *p* < 0.05 is considered significance. For non-obese group, n = 12; for obese group, n = 11. Values are mean ± SEM.

UPR upregulates the expression of C/EBP Homologous Protein (CHOP) and chaperone genes. We found that CHOP was significantly elevated (Figure 4) (Szegezdi et al., 2006; Dong et al., 2018). CHOP has been known to involve ER-stress-induced pathology and cell apoptosis (Hu et al., 2018). The chaperone genes function as mediator for the post-transcriptional modifications and post-translation modifications of newly translated proteins (Ni and Lee, 2007). In this study, we evaluated CHOP and 10 chaperone genes by RT-QPCR and found that CHOP and nine chaperones were upregulated in the obese placenta than the non-obesity (Figure 4). These upregulated chaperone genes include Calnexin, GRP94/gp96, protein disulfide isomerase family A member 6 (PDIA6), calreticulin, glucose regulated protein-58 kDa (GRP58) or ERp57, suppressor of Ire1 and Lhs1 deletion one factor (SIL-1), P58IPK, homocysteine-induced endoplasmic reticulum protein, isoform A (Herp), ERAD-enhancing α-mannosidase-like proteins (EDEM), and ERp72 (Ni and Lee, 2007). Among the chaperone genes we analyzed, only Bip (GRP78) had similar expression levels in both non-obese and obese samples (Figures 3B–D).

## Discussion

We showed that trophoblast is sensitive to ER stress and UPR activation in the obese placenta, which was indeed limited to the IRE1α-XBP1 signaling pathway. Our results reveal that IRE1α was significantly increased at the protein levels compared to those non-obese controls. Furthermore, we found that maternal obesity induces a significant increase of the splice form of XBP1 at both mRNA and protein levels, and a set of UPR chaperon proteins including CHOP, calnexin, EDEM, calreticulin, GPR94, GPR58, p58IPK, Herp, PDIA6, and SIL-1 at mRNA levels. In contrast, no significant difference was observed in p-PERK signaling between the obese and non-obese control. Collectively, this study establishes that ER stress and UPR activation in the placenta is molecular characteristics of maternal obesity.

Our study supports that maternal obesity-induced specific activation of IRE1α-XBP1 plays a critical role in placental pathophysiology associated with obesity. All three UPR conserved signaling pathways share three domains: ER luminal domain, cytosolic domain, and pass membrane spanning domain, which are organized to enable proteins crossing ER membrane into the cytosol (Adams et al., 2019). The cytosolic part of IRE1α harbors kinase domains and can be autophosphorylated, which in turn stimulates the endoribonuclease activity and splices XBP1 to form a potent transcription activator, XBP1s (Cox and Walter, 1996; Calfon et al., 2002). XBP1s-induced upregulation of UPR target chaperone genes enhances a cell’s capacity to assist in protein maturation as well as promotes protein degradation and transportation, thereby releasing the burden of protein misfolding within the ER (Lee et al., 2003). PERK signaling is involved in regulation of the translation response of the UPR. PERK activation can phosphorylate eukaryotic translation initiation factor-2α (EIF2α), a key component of EIF2 complex, and causes ribosome inhibition and a transient attenuation of global protein translation (Harding et al., 2000), which subsequently alleviates the consequences because of protein misfolding to the cellular homeostasis. In this study, we were not able to identify the effects posed by maternal obesity on activity of PERK signaling in the placenta. While the exact mechanism responsible for such differential stimulatory effects remains elusive, obviously, it deserves further investigations.

Multiple studies have shown that ER stress is implicated in a variety of adverse pregnancy outcomes, suggesting that it may constitute a molecular mechanism underlying such associations. Indeed, UPR has been found to account for pathological changes in the placenta in patients complicated with preeclampsia (Yung et al., 2014; Yung et al., 2019; Thomson et al., 2021), miscarriage (Tang et al., 2021), stillbirth and preterm birth (Kawakami et al., 2014). Adverse pregnancy outcomes including preeclampsia are frequently associated with an enhanced apoptosis and stress. To this end, we speculate that UPR activation in the placenta may constitute an important mechanism accounting for maternal obesity-associated adverse pregnancy outcomes.

Our further efforts would include recapitulating pathophysiology of maternal obesity-induced effects on ER stress effects and UPR activation *in vivo*. There are multiple models of causing obesity in animals, which are classified as both genetic including monogenic, polygenic, and transgenic models and non-genetic including dietary, exotic, and large animals (Suleiman et al., 2020). Animal models have been widely used for revealing underlying causes for obesity, which are exemplified by environmental, hereditary, physiological, and epigenetic factors. These obesity animal models will provide an extremely useful model for dissecting molecular signaling pathways including ER stress and UPR responsible for maternal obesity-associated clinical manifestations of adverse obstetrical outcomes.

In conclusion, this is the first report for ER stress and UPR activation in the placenta of maternal obesity. Our findings represent the first step in understanding of one of key ER signaling pathway, also referred to IRE1α-XBP1, in placental pathophysiology affected by obesity, which may be an important mechanism accounting for the observed higher maternal and perinatal risks. Future studies are needed to confirm these findings *in vivo* animal studies and explore the value of p-IRE1α and XBP1s as important biomarkers to predict the adverse pregnancy outcomes (APOs) because of maternal obesity. Finally, we will assess any modifiable exposures that may reduce ER stress and UPR activation in the placenta, which can be largely facilitated by development of selective chemical compounds targeting genes constituting the IRE1α-XBP1 signaling pathway, thus improving clinical outcomes of maternal obesity and its associated APOs while providing individualized medicine.

## Data Availability

The original contributions presented in the study are included in the article/supplementary material, further inquiries can be directed to the corresponding author.
